# High expression of cuproptosis-related SLC31A1 gene in relation to unfavorable outcome and deregulated immune cell infiltration in breast cancer: an analysis based on public databases

**DOI:** 10.1186/s12859-022-04894-6

**Published:** 2022-08-22

**Authors:** Linrong Li, Lin Li, Qiang Sun

**Affiliations:** 1 Department of Breast Surgery, Peking Union Medical College Hospital, Peking Union Medical College, Chinese Academy of Medical Sciences, Beijing, People’s Republic of China; 2 Department of Joint and Orthopedics, Zhujiang Hospital, Second Clinical Medical College, Southern Medical University, Guangzhou, People’s Republic of China

**Keywords:** SLC31A1, Breast cancer, Immune cells, Cuproptosis, Prognosis

## Abstract

Cuproptosis induction represents a promising alternative for immunotherapies and targeted therapies in breast cancer. This study aimed to investigate the prognostic and biological significance of cuproptosis-related genes in breast cancer. In the current study, we examined the transcriptional and clinical data of 13 cuproptosis-related genes in patients with breast cancer from TCGA database. We found that genes DLAT, SLC31A1, ATP7A and ATP7B were significantly related to the overall survival (OS) of breast cancer patients in univariate Cox regression analysis. Unlike lung or kidney cancers, SLC31A1 expression was upregulated in breast cancer samples compared with normal tissues, and predicted poor prognosis. Univariate and multivariate Cox regression analyses indicated that high SLC31A1 level was an independent prognostic factor for shorter OS. A nomogram integrating SLC31A1, age, T-, N-stage and clinical stage was constructed, and the calibration curves of the 1-, 3-, 5-, 10-year OS fitted well with the ideal model. Furthermore, we found that high SLC31A1 expression was related to deregulated immune response and metabolic pathways. Low SLC31A1 level predicted sensitivity to CTLA4 inhibitors but poor response to paclitaxel. Our study may provide novel insights for copper homeostasis and cuproptosis in breast cancer.

**Supplementary Information:**

The online version contains supplementary material available at 10.1186/s12859-022-04894-6.

## Background

Breast cancer is the most frequently diagnosed cancer [1], and ranks first among all causes of cancer-related death in women worldwide [2]. Breast cancer is a heterogeneous disease with distinct molecular features, biological characteristics and clinical outcomes [3]. Despite advances in surgery, endocrine therapy, chemotherapy and radiotherapy, patients with metastatic breast cancer expected a five-year relative survival rate of 29% [4]. And for those with metastatic triple negative breast cancer (TNBC), the median progression-free survival (PFS) was less than 8 months [5, 6].

Recently, advances in immunotherapy have profoundly improved the clinical outcomes of TNBC, represented by PD-L1 (programmed death ligand 1) inhibitor atezolizumab and PD-1 (programmed death 1) inhibitor pembrolizumab [7, 8]. According to final results from the KEYNOTE⁃355 study, pembrolizumab in addition to chemotherapy significantly improved PFS in patients with advanced TNBC whose tumors expressed PD-L1 (9.7 vs 7.6 months, hazard ratio, HR 0.66 [95% confidential interval, CI, 0.50–0.88]) [8]. Meanwhile, targeted therapies have become a standard treatment option in breast cancer. Patients with human epidermal growth factor 2 (HER2)-positive advanced breast cancer expected a median PFS of 18.5 months in the era of HER2-targeted agents trastuzumab and pertuzumab [9, 10]. However, immunotherapy and targeted therapy were greatly limited by low response rates, lack of effective response predictors, resistance as well as drug toxicity [11–13]. In this context, therapies which could induce alternate forms of regulated cell death, ferroptosis, pyroptosis, necroptosis and apoptosis, for instance, become potential strategies to eliminate resistant breast cancer cells and improve survival [11].

Copper, as an essential cofactor for a variety of metalloenzymes that contribute to tumor metastasis [14], has recently become a rising topic of research in the development of antitumor therapies. Study showed copper homeostasis dysregulation could induce proteotoxic stress and cell death by direct binding to lipoylated tricarboxylic acid cycle proteins [15]. A total of 13 genes were related with cuproptosis according to Tsvetkov P. et al., including FDX1, LIPT1, LIAS, DLD, DBT, GCSH, DLST, DLAT, PDHA1, PDHB, SLC31A1 (solute carrier family 31 member 1), ATP7A and ATP7B [15]. Accordingly, cuproptosis induction represents an upcoming alternative for the treatment of breast cancer. However, the prognostic and biological significance of copper homeostasis in breast cancer have yet to be fully elucidated.

**Fig. 1:**
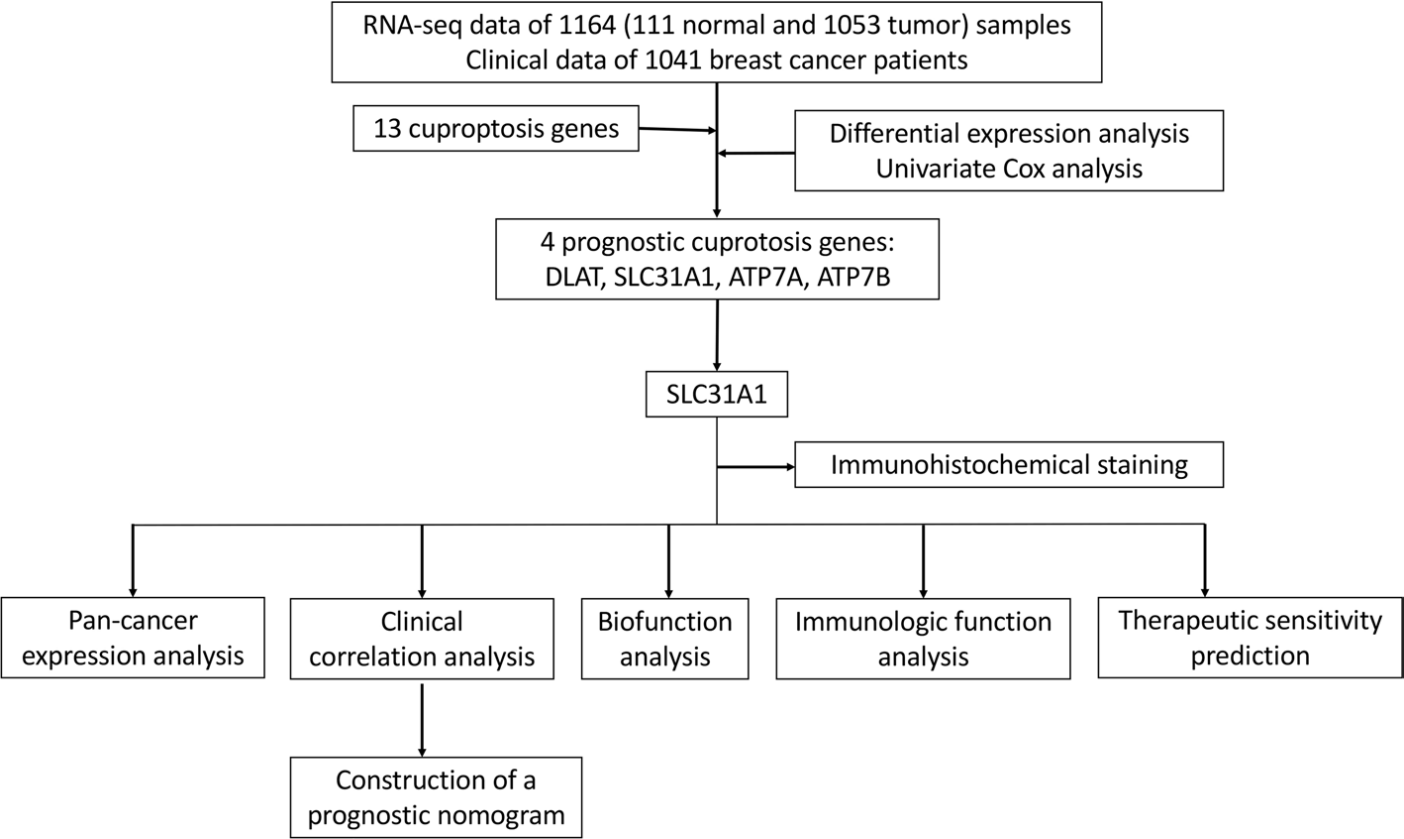
Flow diagram of the study

In our study (Fig. 1), we analyzed the expression and prognostic profiles of 13 cuproptosis-related genes in breast cancer samples downloaded from the Cancer Genome Atlas (TCGA) database. The expression level of SLC31A1 was found to be higher in the breast cancer samples compared with normal tissues. High SLC31A1 expression correlated with a poor prognosis in patients with breast cancer, compared with low SLC31A1 expression. Pan-cancer expression profiles of SLC31A1 were also provided. Next, we developed a nomogram based on SLC31A1 expression, age, T-, N-stage and clinical stage to predict the 1-, 3-, 5- and 10-year overall survival (OS) of patients with breast cancer. To study the underlying molecular mechanisms, SLC31A1-related genes, pathways and cellular functions were explored with Gene Ontology (GO), Kyoto Encyclopedia of Genes and Genomes (KEGG) enrichment and gene set enrichment analysis (GSEA). Using ‘CIBERSORT’, we found that SLC31A1 expression was associated with deregulated tumor-infiltrating immune cells. Also, correlations between SLC31A1 and drug sensitivities were explored utilizing Cancer Immunome Atlas (TCIA) and TCGA database. Low SLC31A1 expression was related with poor response to paclitaxel but sensitivity to CTLA4 inhibitors. Hence, it is plausible that SLC31A1 overexpression induces copper homeostasis dysregulation, thus possibly interfering with antitumor immune effects in breast cancer. SLC31A1 exhibits a promising therapeutic target for cuproptosis-induction and combined therapy in breast cancer.

## Results

### Cuproptosis-related gene SLC31A1 was deregulated in cancers and related to prognosis in breast cancer

**Fig. 2:**
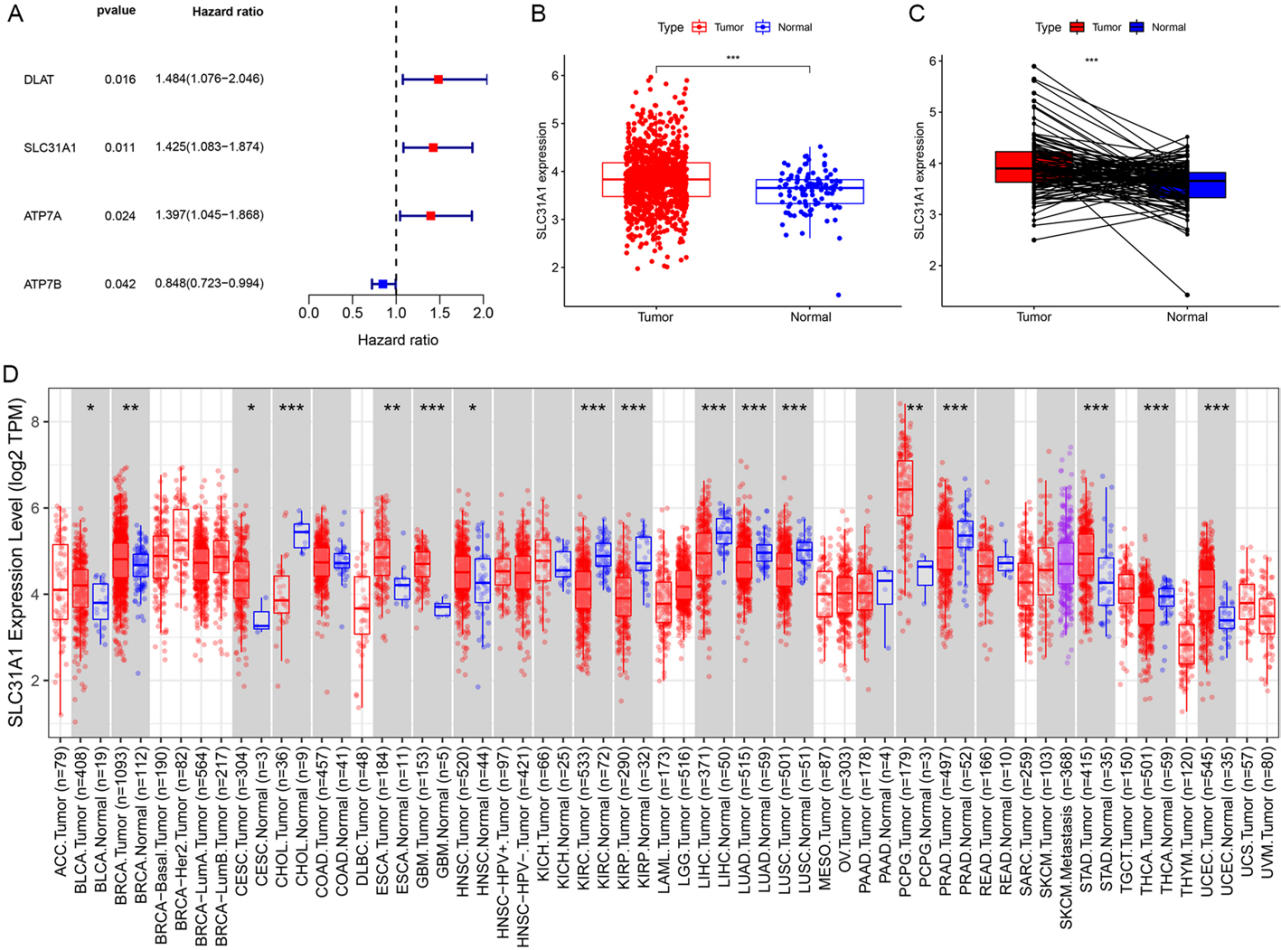
Identification of prognostic cuproptosis-related genes in breast cancer. **A** 4 cuproptosis-related genes, DLAT, SLC31A1, ATP7A and ATP7B, were correlated with prognosis of breast cancer patients. **B** Expression of SLC31A1 in tumor samples was higher than that in normal samples in breast cancer. **C** For each patient, tumor tissues generally showed higher expression levels of SLC31A1 than normal tissues, based on data from TCGA database. Y-axis represents log2 (TPM) values of SLC31A1 expression of samples. **D** Pan-cancer expression analysis of SLC31A1 between normal and tumor samples according to TIMER. BLCA, bladder urothelial carcinoma; BRCA, breast invasive carcinoma; CESC, cervical squamous cell carcinoma and endocervical adenocarcinoma; ESCA, esophageal carcinoma; GBM, glioblastoma multiforme; HNSC, head and neck squamous cell carcinoma; PCPG, pheochromocytoma and paraganglioma; STAD, stomach adenocarcinoma; UCEC, uterine corpus endometrial carcinoma; CHOL, cholangiocarcinoma; KIRC, kidney renal clear cell carcinoma; KIRP, kidney renal papillary cell carcinoma; LUSC, lung squamous cell carcinoma; prostate adenocarcinoma; THCA, thyroid carcinoma; *P < 0.05, **P < 0.01, ***P < 0.001

Gene expression quantification data of transcriptome profiling (HTseq-FPKM) of 1164 (including 1053 tumor and 111 normal) samples of breast cancer together with correlated clinical information of 1041 breast cancer patients were downloaded from TCGA database. All 13 cuproptosis-related genes were differentially expressed between the breast cancer samples and normal samples (Additional file 1: Figure S1). Gene ATP7B, SLC31A1 and PDHB were higher expressed in the tumor samples, while gene ATP7A, PDHA1, DBT, DLAT, DLD, DLST, FDX1, GCSH, LIAS and LIPT1 were higher expressed in the normal samples. Among them, expression levels of genes DLAT, SLC31A1, ATP7A and ATP7B were found to be significantly related to OS in univariate Cox regression analysis (Fig. 2A, *P* = 0.016, 0.011, 0.024, 0.042, respectively). Based on its smallest P value in univariate Cox regression as well as Kaplan–Meier (K-M) analysis (Additional file 1: Figure S2), SLC31A1 was chosen to be further investigated. The expression levels of SLC31A1 of breast cancer samples were significantly higher than those of normal samples (Fig. 2B). For each patient, SLC31A1 expression of the tumor sample was higher than that of the normal sample (Fig. 2C). Results from the pan-cancer expression analysis of SLC31A1 were demonstrated (Fig. 2D). In addition to breast cancer, higher levels of SLC31A1 were found in tumor samples of bladder, cervical, esophageal, uterine, stomach, head and neck cancers, as well as pheochromocytoma and paraganglioma, compared with normal samples. On the contrary, SLC31A1 was found to be higher in normal samples compared with tumor samples of cholangiocarcinoma, kidney, lung, prostate and thyroid cancers.

### Validation of the prognostic value of SLC31A1 in breast cancer

**Fig. 3:**
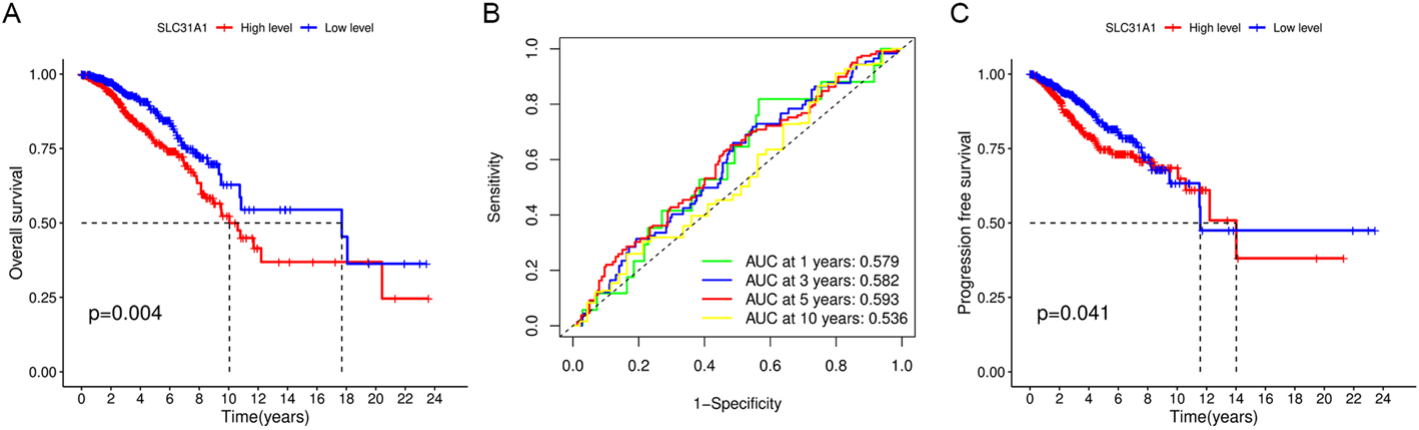
**A** Kaplan–Meier survival curves for overall survival in breast cancer patients according to the tumor expression of SLC31A1. **B** 1-, 3-, 5-, and 10-year time dependent receiver operating characteristic curves of the predictive value of SLC31A1 for overall survival. **C** Kaplan–Meier survival curves for progression-free survival in breast cancer patients according to the tumor expression of SLC31A1

After excluding 7 patients with no SLC31A1 expression data (n = 6) or OS time (n = 1), a total of 1034 patients with clinical data were included for survival analysis (Table 1). According to the median cut-off value, 517 patients with breast cancer were divided into high and low SLC31A1 expression groups, respectively. Results from the survival analysis displayed that OS of the low expression group was significantly longer than that of the high expression group (Fig. 3A, *P* = 0.004). The area under curves (AUCs) of the SLC31A1 expression groups for OS at 1, 3, 5, and 10 years were 0.579, 0.582, 0.593 and 0.536, respectively (Fig. 3B). In addition, PFS of the low expression group was significantly longer than that of the high expression group (Fig. 3C, *P* = 0.041).

**Table 1:** Clinical characteristics of the 1034 breast cancer patients from the Cancer Genome Atlas database

Clinical factor	
Age (mean ± SD [min, max])	58.21 ± 13.21 [26,90]
Overall survival time (mean ± SD [min, max])	1250 ± 1202 [0, 8605]
Survival status	
Living	893 (86.36%)
Dead	141 (13.64%)
Missing	0 (0%)
Pathologic T	
T1	268 (25.92%)
T2	600 (58.03%)
T3	128 (12.38%)
T4	35 (3.38%)
Missing	3 (0.29%)
Pathologic M	
M0	860 (83.17%)
M1	21 (2.03%)
Missing	153 (14.80%)
Pathologic N	
N0	477 (46.13%)
N1	345 (33.37%)
N2	118 (11.41%)
N3	74 (7.16%)
Missing	20 (1.93%)
Pathologic stage	
I	171 (16.54%)
II	587 (56.77%)
III	234 (22.63%)
IV	19 (1.84%)
Missing	23 (2.22%)

### Correlation between SLC31A1 and other clinical characteristics

**Fig. 4:**
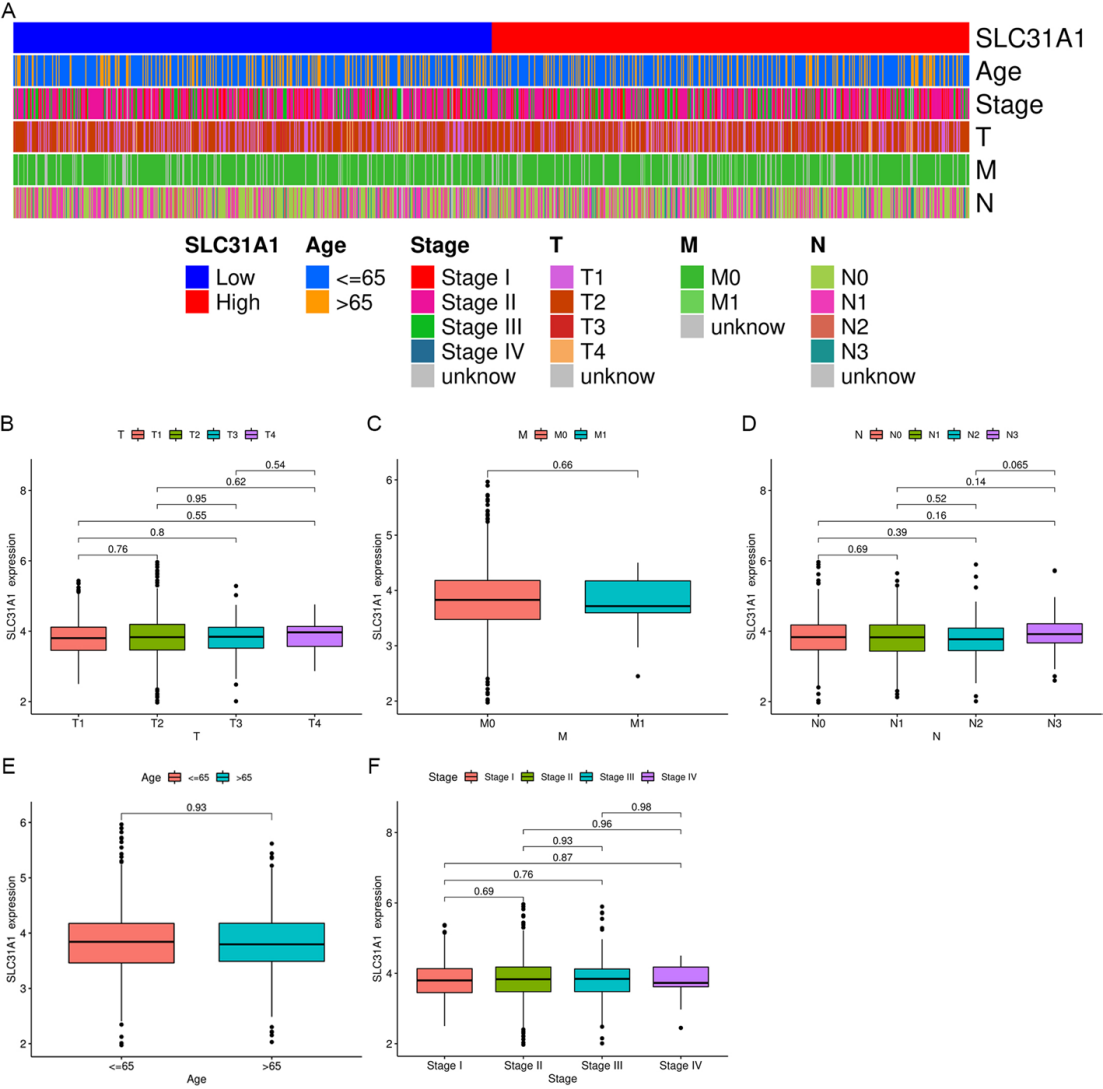
Correlation between SLC31A1 and clinical characteristics. **A** Heatmap showing distribution of clinical characteristics between high and low SLC31A1 expression groups. Differential expression of SLC31A1 for breast cancer patients according to clinical characteristics are presented: **B** T-stage, **C** M-stage, **D** N-stage, **E** age, **F** clinical stage

There was no statistical significance between SLC31A1 and clinical characteristics including age, clinical stage, T-, M- and N-stage (Fig. 4).

### Construction and validation of a prognostic nomogram

**Fig. 5:**
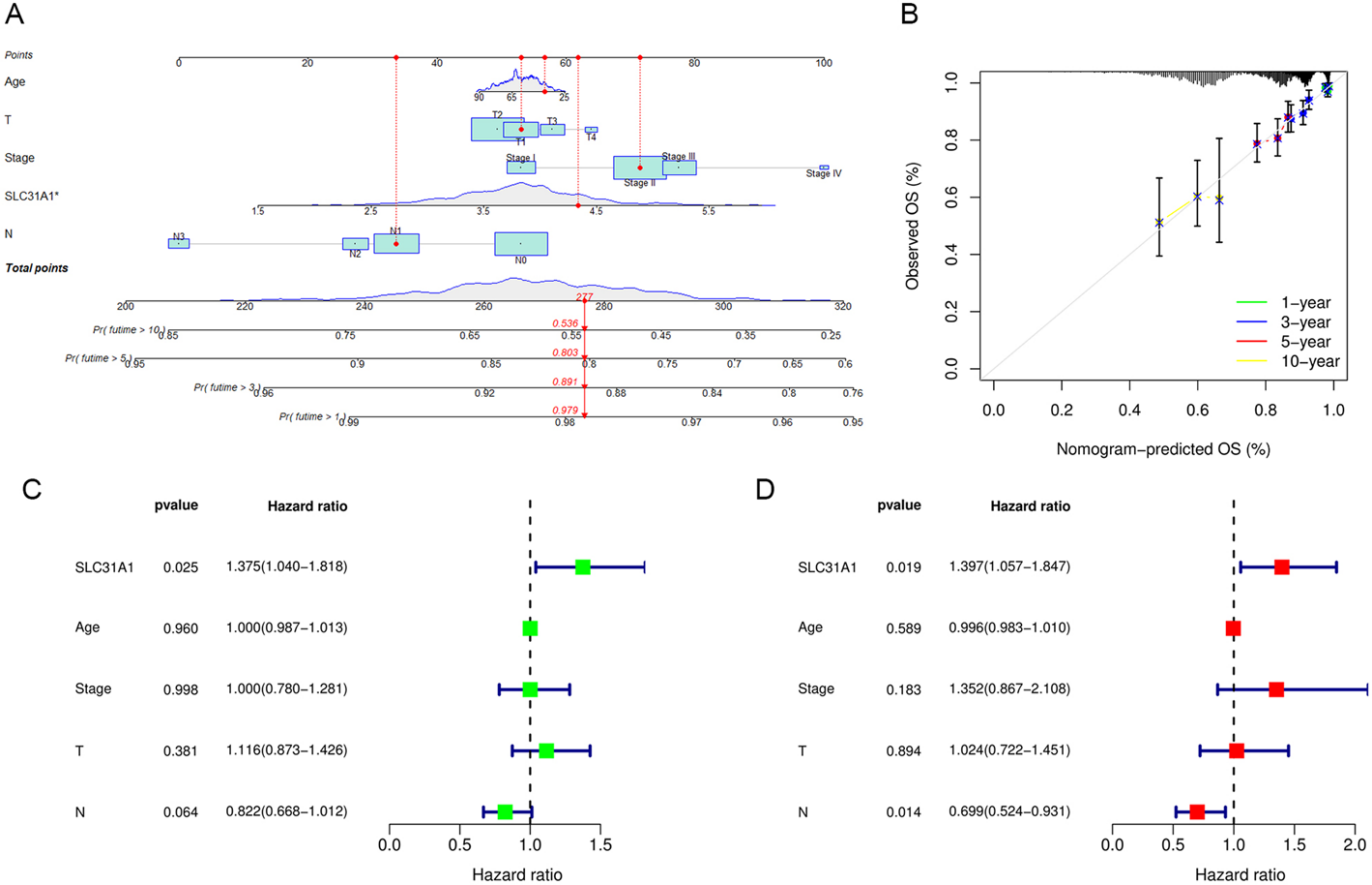
Construction and validation of a prognostic nomogram. **A** Nomogram for overall survival prediction, with age, T-stage, N-stage, clinical stage, and the expression level of SLC31A1 applied as parameters. **B** Calibration curves of the nomogram for 1-, 3-, 5-, 10-year overall survival prediction. **C** Univariate and **D** multivariate Cox regression analysis identified SLC31A1 as an independent clinical characteristic for overall survival prediction

A nomogram was established for 1-, 3-, 5-, and 10-year OS prediction in breast cancer based on TCGA data. The expression level of SLC31A1, age, T-, N-stage and clinical stage were eventually applied as parameters (Fig. 5A). M-stage was excluded due to statistical uncertainty and imbalanced distribution. The calibration curves of the 1-year, 3-year, 5-year, 10-year OS fitted well with the ideal model (Fig. 5B). We next conducted univariate and multivariate Cox regression analyses to examine the independent prognostic role of SLC31A1 in breast cancer with other clinical characteristics, including age, T-stage, N-stage and clinical stage. Univariate and multivariate regression analyses showed that the SLC31A1 was an independent prognostic factor for OS with a HR of 1.375 (95% CI, 1.040–1.818) and 1.397 (95% CI, 1.057–1.847), respectively (Fig. 5C, D).

### Identification of related genes, pathways and cellular functions of SLC31A1 in breast cancer

**Fig. 6:**
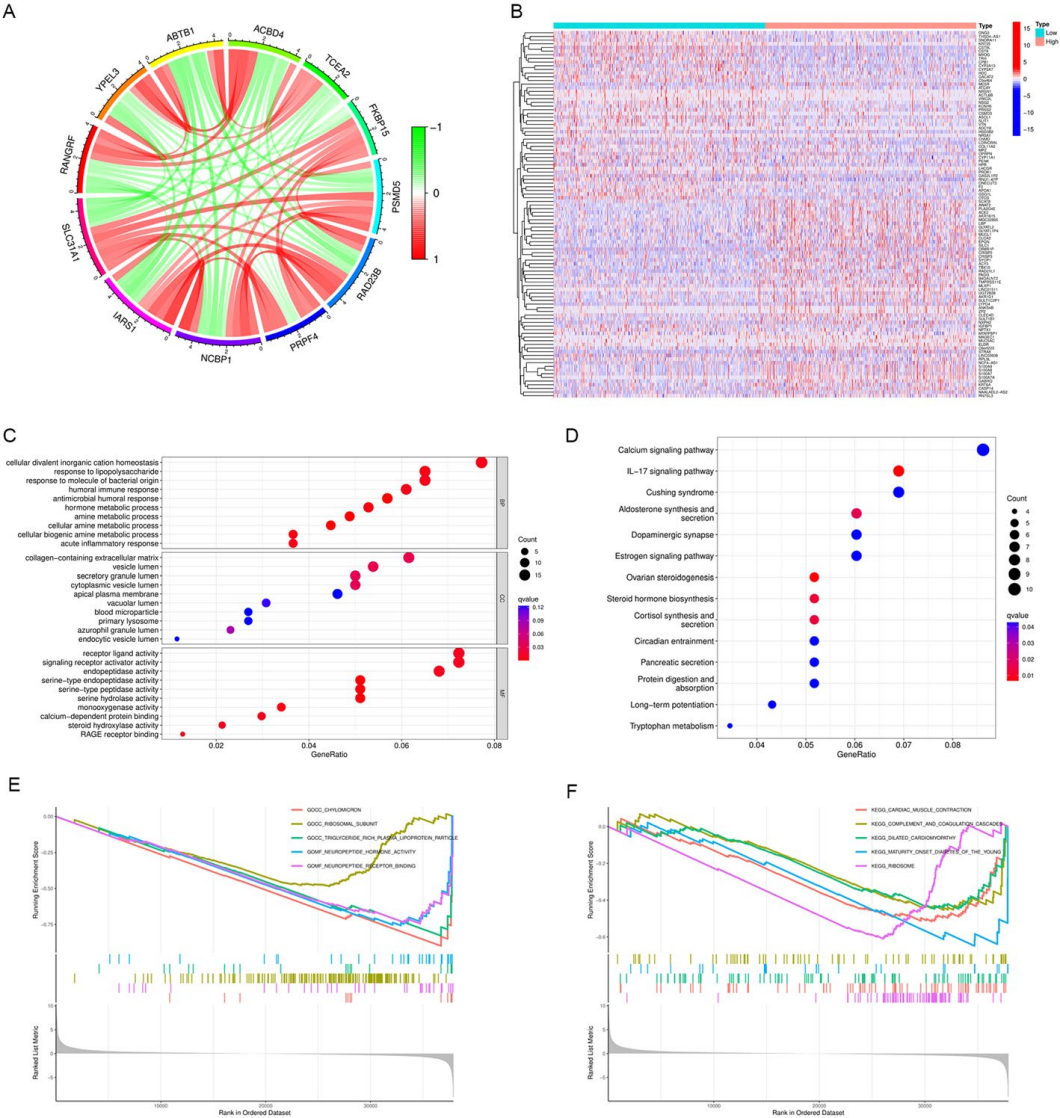
Identification of related genes, pathways and cellular functions of SLC31A1. **A** Circos graph displaying the co-expression networks of SLC31A1 with 11 genes in breast cancer samples. Each sector of the circle represents one gene, and its width indicates the total amount of co-occurrence that connects one certain gene to the other. The width of each link represents the total co-expression times of the linked genes. **B** Heatmap showing 186 down-regulated genes (blue) and 164 up-regulated genes (red) identified in the high expression group. The colored matrix shows hierarchical clustering of expression microarrays, with each column representing the log2 (TPM) values of expression levels of 350 genes in microarray hybridization of one sample, and each row representing the expression of a particular gene described to the right. **C** Results from Gene Ontology (GO) analysis. Genes of the immune response and metabolic process were mostly enriched. **D** Results from Kyoto Encyclopedia of Genes and Genomes (KEGG) enrichment analysis. Genes of the calcium signaling pathway and IL-17 signaling pathway were mostly enriched. **E** GSEA analysis revealed down-regulated pathways associated with SLC31A1 expression. The GSEA analysis of GO between the high SLC31A1 expression samples and low SLC31A1 expression samples revealed significant difference in the enrichment of chylomicron and triglyceride pathways. **F** The GSEA analysis of KEGG pathway enrichment between the high SLC31A1 expression samples and low SLC31A1 expression samples revealed significant difference in the enrichment of dilated cardiomyopathy, cardiac muscle contraction and ribosome pathways

Based on the RNA sequencing data from TCGA database, co-expression analysis was performed and a total of 206 genes were found to be significantly correlated with SLC31A1, 55 negatively and 151 positively. Figure 6A demonstrated the interaction among SLC31A1 and eleven genes highly related with SLC31A1. Furthermore, a total of 350 differentially expressed genes (DEGs) were identified between high and low SLC31A1 expression groups, of which 164 genes were up-regulated and 186 genes were down-regulated in the high expression group (Fig. 6B). GO analysis and KEGG pathway analysis were conducted, and the DEGs were found to be mainly enriched in immune response and metabolic process (Fig. 6C), together with calcium and IL-17 signaling pathways (Fig. 6D). In addition, GSEA revealed that cellular functions associated with metabolism and cardiomyopathy were enriched in breast cancer patients with low SLC31A1 expression (Fig. 6E, F).

### Correlation between SLC31A1 and immune cell infiltration in breast cancer

**Fig. 7:**
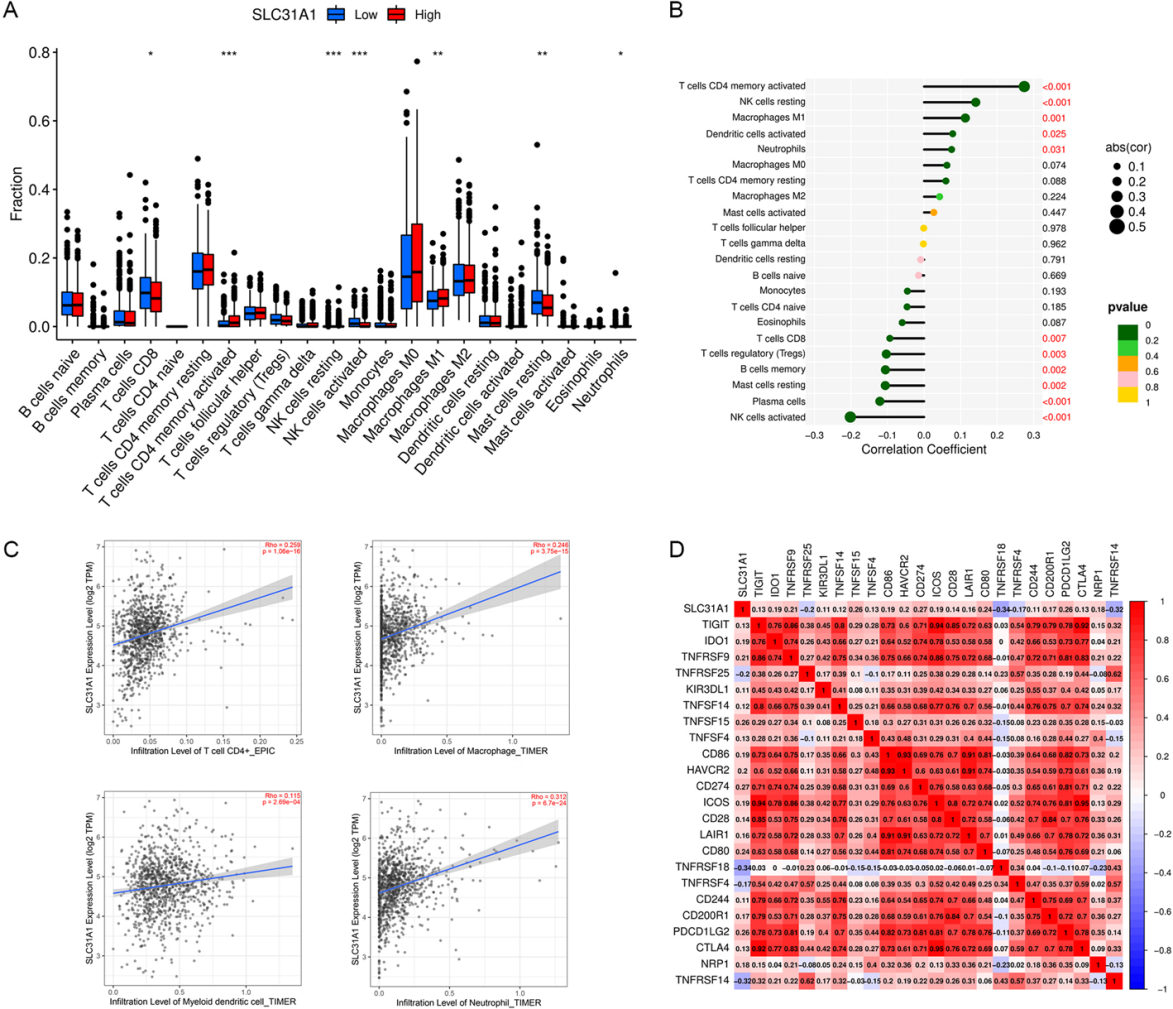
Correlation between SLC31A1 and immune infiltration. **A** The infiltration levels of activated memory CD4 T cells and macrophages M1 were upregulated in high SLC31A1 expression group, while the infiltration levels of CD8 T cells and activated NK cells were downregulated in the same group. **B** Lollipop graph showing the correlation between immune infiltration and SLC31A1 expression. The infiltration level of activated CD4 memory T cells was positively correlated with SLC31A1, while the infiltration level of activated NK cells was negatively correlated with SLC31A1. **C** Scatter plots showing the correlation between the expression levels of SLC31A1 and immune infiltration according to TIMER. The expression level of SLC31A1 was positively related with the infiltration level of CD4 T cell, macrophage, myeloid dendric cell, and neutrophil. **D** Correlation between SLC31A1 and immune checkpoint-related genes. TNFRSF18 and TNFRSF14 were negatively related with SLC31A1, while TNFSF15, CD274, CD80 and PDCD1LG2 were positively related with SLC31A1

Differential analysis of immune cell infiltration levels between high and low SLC31A1 expression groups was performed based on ‘CIBERSORT’. Significant correlation was demonstrated between SLC31A1 and tumor-infiltrating immune cells. The infiltration levels of activated memory CD4 T cells, natural killer (NK) cells resting, macrophages M1, activated dendritic cells and neutrophils were positively correlated with SLC31A1, while the infiltration levels of CD8 T cells, regulatory T cells, memory B cells, resting mast cells, plasma cells and activated NK cells were negatively correlated with SLC31A1 (Fig. 7A, B). According to TIMER, the expression level of SLC31A1 was positively related with CD4 + T cells, macrophages, myeloid dendric cells and neutrophils (Fig. 7C).

### Correlation between SLC31A1 and drug sensitivity in breast cancer

**Fig. 8:**
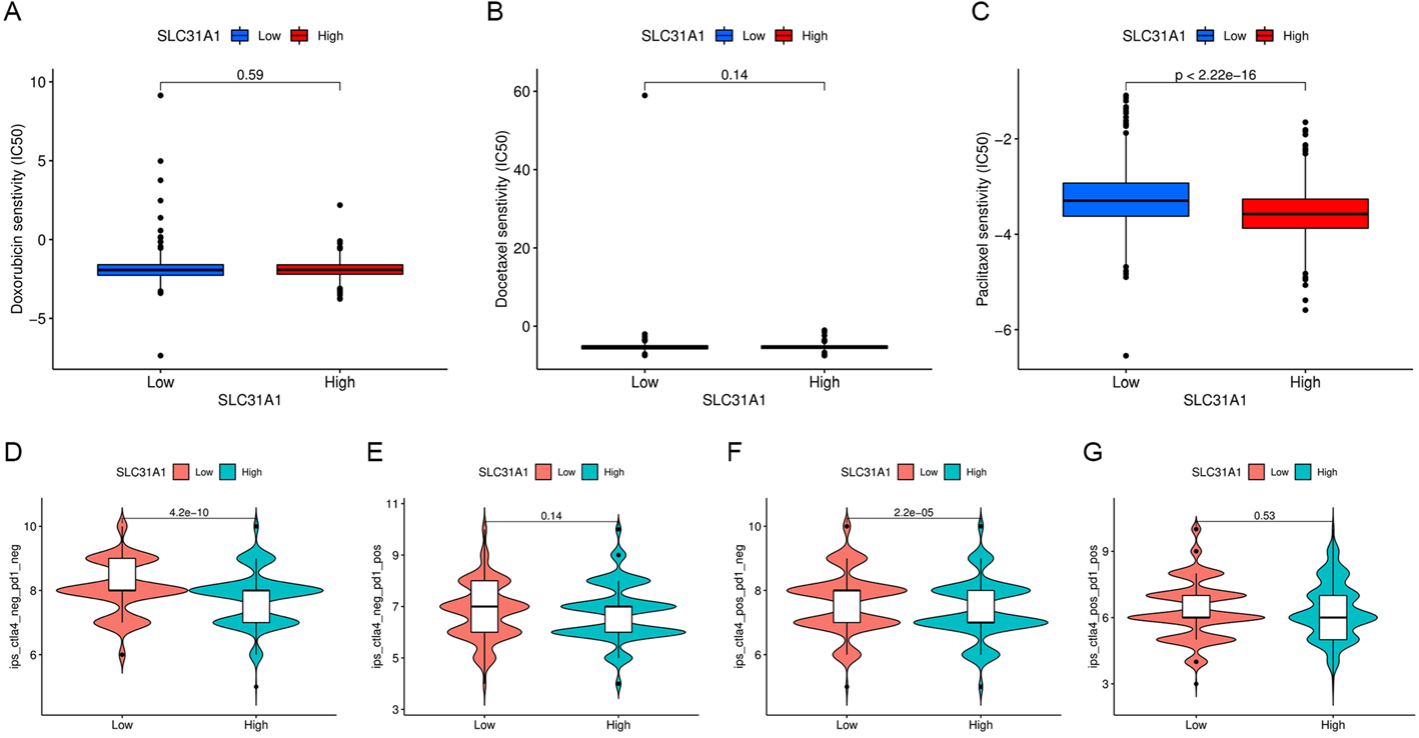
Therapeutic sensitivity prediction with SLC31A1 in breast cancer. Comparison of the half maximal inhibitory concentration of anti-tumor drugs between high and low SLC31A1 expression groups: **A** doxorubicin, **B** docetaxel and **C** paclitaxel. Immune therapy scores (vertical axis) of anti-CTLA4 and anti-PD1 inhibitors indicating response of **D** no immunotherapy, **E** anti-PD1, **F** anti-CTLA4, **G** anti-PD1 and anti-CTLA4 therapies between high and low SLC31A1 expression groups

First, we explored the correlation between SLC31A1 and immune checkpoint inhibitors using expression levels of SLC31A1 and 23 immune checkpoint-related genes. SLC31A1 was significantly related with all 23 immune checkpoint-related genes (*P* < 0.001). Among them, SLC31A1 was negatively related with TNFRSF18 and TNFRSF14, while positively related with TNFSF15, CD274, CD80 and PDCD1LG2 (Fig. 7D). Therapy scores of anti-CTLA4 and anti-PD1 inhibitors were also obtained, with higher therapy scores indicating better therapeutic outcomes. Statistical significance was found only in the CTLA4-positive-PD1-negative section (Fig. 8D–G), suggesting that anti-CTLA4 therapy rather than anti-PD1 therapy was better choice for patients with low SLC31A1 expression. Next, differential analysis of half maximal inhibitory concentration (IC50) of common chemotherapy drugs including doxorubicin, docetaxel and paclitaxel between high and low SLC31A1 expression groups was conducted. The IC50 of paclitaxel was significantly lower in the high expression group (Fig. 8C, *P* < 0.001). No significant correlation was found between SLC31A1 and doxorubicin or docetaxel (Fig. 8A, B). The results indicated that high SLC31A1 expression in breast cancer predicted better response to paclitaxel, but not doxorubicin or docetaxel.

### Immunohistochemical staining

**Fig. 9:**
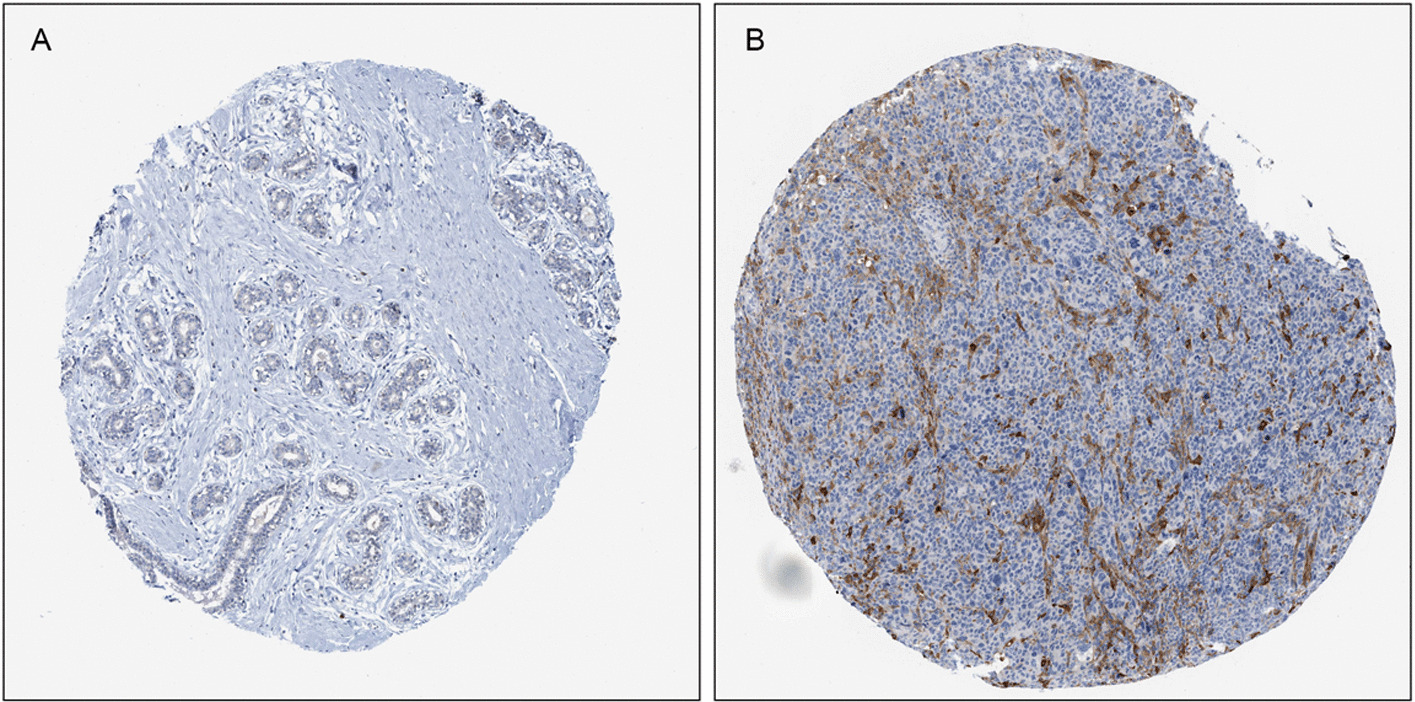
Representative immunohistochemical staining for SLC31A1 in **A** normal breast tissue and **B** breast cancer tissue, taken from the Human Protein Atlas with permission on its website. HPA013810/SLC31A1/data available from v21.0. https://www.proteinatlas.org/ENSG00000136868-SLC31A1/tissue/breast#img (**A**); HPA013810/SLC31A1/data available from v21.0. https://www.proteinatlas.org/ENSG00000136868-SLC31A1/pathology/breast+cancer#img (**B**)

Immunohistochemical staining figures of normal breast tissues and tumor tissues were obtained from the Human Protein Atlas (HPA) database. Figures showed that the expression level of SLC31A1 in the breast tumor tissue was remarkably higher than that in the normal tissue (Fig. 9A, B).

## Discussion

SLC31A1, also named as CTR1 (copper transporter 1), is a major copper importer that exerts important functions in intracellular copper homeostasis [16]. SLC31A1 overexpression led to an increase in copper uptake in breast cancer cells and xenograft models [17, 18]. Copper is a double-edged sword. On the one hand, stable intracellular copper concentration is essential to the function of specific enzymes to induce pro-angiogenic response and activate metabolism and tumor proliferation [19, 20]. This is consistent with our findings of GO, KEGG and GSEA analysis that SLC31A1 was closely related with metabolism. Furthermore, our study showed that low levels of SLC31A1 in breast cancer were associated with favorable clinical outcomes, which is probably through copper depletion. Copper depletion with the oral copper chelating agent tetrathiomolybdate (TM) blocked TNBC metastasis through inactivation of Complex IV and reduced mitochondrial oxidative adenosine triphosphate production [21]. Previous results from the phase II study of TM reported a 69% rate of event-free survival at a median follow-up of 6.3 years in stage IV TNBC without any evidence of disease [22].

On the other hand, excess copper resulted in cuproptosis [15]. By fostering accumulation of intracellular free copper, SLC31A1 was considered as a key molecule promoting cuproptosis in kidney and lung cancer cell lines [15]. A study for non-small-cell lung cancer (NSCLC) revealed that SLC31A1 rs10759637 variant at 3ʹUTR led to decreased levels of SLC31A1 and shorter OS (HR 1.24; *P* = 0.005) [23]. This seems contrary to our results. The seemingly conflicting data could probably be explained by the following aspects. First, SLC31A1 was also important for the uptake of platinum due to its low-affinity, and considered important for tumor response to platinum drugs [24, 25]. Second, platinum-based chemotherapy was widely applied in NSCLC, and the reduced uptake of platinum resulting from SLC31A1 downregulation could reduce drug sensitivities and clinical outcomes. However, platinum is not as widely used in breast cancer, which is only an option in the treatment of advanced breast cancer and TNBC, etc. [26]. Last but not the least, the mechanisms of cuproptosis have not been validated in breast cancer. Whether the accumulation of intracellular copper with high levels of SLC31A1 caused proteotoxic stress and cell death in breast cancer remains to be validated. Rather, intracellular copper depletion could suppress breast cancer, as mentioned above. Our pan-cancer expression analysis results also showed that SLC31A1 was upregulated in breast cancer samples, while downregulated in kidney and lung cancer samples. Therefore, SLC31A1 upregulation and intracellular copper accumulation represent a possible mechanism for breast cancer tumorigenesis and progression. Further research is required to elucidate the underlying mechanisms of copper homeostasis and cuproptosis in breast cancer.

Tumor microenvironment, composed of malignant cells, fibroblasts, and tumor-infiltrating immune cells, play an essential role in tumorigenesis, and display important prognostic value for breast cancer [27]. It was demonstrated that high levels of tumor-infiltrating lymphocytes (TILs) in breast cancer were associated with significantly improved OS compared with low levels of TILs, irrespective of subtype [28]. Copper supplementation was reported to enhance PD-L1 expression in malignant cells. Conversely, copper-chelating drugs induced ubiquitin-mediated degradation of PD-L1, increased the number of CD8 T and NK cells, inhibited tumor growth, and improved survival [29]. In our research, significant correlations were demonstrated between SLC31A1 and immune infiltration. Consistent with previous research, the infiltration levels of CD8 T cells and activated NK cells were negatively associated with SLC31A1 expression. Anti-CTLA4 therapy rather than anti-PD1 therapy showed higher therapy scores in patients with low SLC31A1 expression.

The present study has certain limitations to be thoroughly considered. First, the statistical analysis was based on data derived from public-available databases. Further experiments are required to validate our results. Second, the molecular subtyping and treatment information were not available for data analyzation, which might have potential influence on the results. Last but not least, though the calibration curves of the SLC31A1-based nomogram fitted well with the ideal model, AUCs of the SLC31A1 expression groups for OS were less than 0.6. Since DLAT, SLC31A1, ATP7A and ATP7B were all found to be related to prognosis in breast cancer, a combined prognostic model aggregating more cuproptosis-related genes and clinical features is expected in future.

## Conclusion

To conclude, we investigated the prognostic and biological role of cuproptosis-related gene SLC31A1 in breast cancer. High expression of SLC31A1 was found to be related with unfavorable outcome and deregulated tumor-infiltrating immune cells. With a better understanding of copper homeostasis, SLC31A1 exhibits a promising biomarker for drug sensitivity and a novel therapeutic target for overcoming drug resistance in breast cancer.

## Methods

### Data processing

RNA sequencing data and correlated clinical information of patients with breast cancer were downloaded from TCGA (https://cancergenome.nih.gov/) database. Clinical data including PFS and OS of patients in TCGA database were downloaded from Xena Functional Genomics Explorer (https://xenabrowser.net/). Included in this study were female patients with breast cancer. Original data were summarized and processed with Strawberry perl (v5.30.0.1). Subsequent analyses were performed with R software (v4.1.1) [30].

### Identification of prognostic cuproptosis-related genes in breast cancer

Differential expression analysis of 13 cuproptosis-related genes between normal and tumor samples was performed via paired t-tests, including FDX1, LIPT1, LIAS, DLD, DBT, GCSH, DLST, DLAT, PDHA1, PDHB, SLC31A1, ATP7A and ATP7B. We then performed univariate Cox regression analysis in order to find prognosis- and cuproptosis- related genes. K-M survival curves were constructed between normal and tumor samples to evaluate whether the prognosis- and cuproptosis- related genes were prognostic factors of OS. The expression levels of SLC31A1 of both normal and tumor samples were demonstrated with boxplots. In addition, we explored pan-cancer expression levels of SLC31A1 between normal and tumor samples using TIMER 2.0 (http://timer.comp-genomics.org) [31]. R packages ‘survival’, ‘survminer’ were applied in the process.

### Validation of the prognostic value of SLC31A1 in breast cancer

According to the median cut-off value, patients with breast cancer were divided into high and low expression groups. Survival analysis was performed, and K-M survival curves were constructed between the two groups to evaluate whether SLC31A1 was a prognostic factor of OS and PFS, respectively. The time dependent receiver operating characteristic curves (ROCs) were constructed to evaluate the predictive value of SLC31A1 for OS. R packages ‘survival’, ‘survminer’ and ‘timeROC’ were applied in the process.

### Correlation analysis between SLC31A1 and other clinical characteristics

*χ*^2^ or Wilcoxon signed-rank test, when appropriate, was conducted to investigate the correlation between the expression level of SLC31A1 and clinical characteristics including age, clinical stage, T-, M- and N-stage. The results were demonstrated by the boxplot and heatmap. R packages ‘ggpubr’ and ‘ComplexHeatmap’ were used in the procedure.

### Construction and validation of a prognostic nomogram

To establish a prognostic model for predicting OS in breast cancer, a nomogram based on the expression level of SLC31A1, age, T-, M-, N-stage and clinical stage was constructed. Then, calibration curves of the 1-year, 3-year, 5-year, 10-year survival rates were drawn to verify the consistency of the OS data. Moreover, univariate and multivariate Cox regression analyses were performed to verify SLC31A1 as an independent clinical characteristic for OS prediction. The ‘survival’, ‘regplot’ and ‘rms’ packages of R software were utilized in the procedure.

### Identification of related genes, pathways and cellular functions of SLC31A1 in breast cancer

To explore the biological function of SLC31A1 in breast cancer, correlation analysis between SLC31A1 and other genes was performed, with thresholds of |cor|> 0.4 and false discovery rate (FDR) < 0.05. Genes that highly correlated with SLC31A1 were illustrated with a Circos graph. Furthermore, differential gene expression analysis between the high and low expression groups was conducted to identify DEGs, with the thresholds determined as |logFC|> 1 and FDR < 0.05. GO [32], KEGG enrichment analysis [33] and GSEA [34, 35] were performed to find enriched molecular mechanisms and cellular functions of the obtained DEGs. R packages ‘ggplot2’, ‘ggpubr’, ‘ggExtra’, ‘circlize’, ‘corrplot’ and ‘pheatmap’ were applied in this process.

### Correlation analysis between SLC31A1 and immune cell infiltration in breast cancer

In order to evaluate the correlation between SLC31A1 and immune cell infiltration in breast cancer, the proportions of tumor‐infiltrating immune cells were calculated using ‘CIBERSORT’, containing the expression features of 22 immune cell subtypes (https://cibersortx.stanford.edu/) [36]. Differences in immune cell infiltration levels between high and low SLC31A1 expression groups were analyzed, and the results were demonstrated with boxplots. Besides, scatter plots were applied to demonstrate the correlation between the expression levels of SLC31A1 and immune cell infiltration using TIMER. The correlation between immune cells and the expression level of SLC31A1 was also presented with a lollipop graph. The ‘reshape2’, ‘ggpubr’, ‘vioplot’ and ‘ggExtra’ packages of R software were utilized in the procedure.

### Correlation analysis between SLC31A1 and drug sensitivity in breast cancer

To predict potential benefits from immunotherapies and chemotherapies in breast cancer patients using SLC31A1, we investigated the expression correlation between SLC31A1 and common drug targets. First, expression correlation analysis between SLC31A1 and immune checkpoint-related genes was performed. Immune therapy scores of samples in TCGA database were obtained from TCIA (https://tcia.at) [37], and the therapy scores for anti-CTLA4 and anti-PD1 inhibitors were compared between the high and low SLC31A1 expression groups. In addition, to predict potential benefits from chemotherapies in patients with breast cancer, the correlation between the expression level of SLC31A1 and the IC50 of common chemotherapeutic drugs including doxorubicin, docetaxel and paclitaxel was investigated, The IC50 data was downloaded from Genomics of Drug Sensibility in Cancer (GDSC, https://www.cancerrxgene.org/). The ‘reshape2’, ‘ggpubr’, ‘ggplot2’, ‘pRRophetic’ and ‘corrplot’ packages of R software were utilized in the procedure.

### Immunohistochemical staining

To validate whether SLC31A1 was differentially expressed between normal tissues and tumor tissues, the immunohistochemical staining figures of both normal breast tissues and breast cancer tissues were obtained from the HPA (https://www.proteinatlas.org/) [38].

## Supplementary Information

**Additional file 1: Figure S1.** Differential expression analysis of 13 cuproptosis-related genes between normal and breast cancer samples. **Figure S2.** Kaplan-Meier plots of 4 prognosis- and cuproptosis- related genes. **Figure S3.** Scatter plots of correlation analysis between SLC31A1 and the other 12 cuproptosis-related genes.

## Data Availability

The datasets analyzed during the current study are available in the Cancer Genome Atlas (https://cancergenome.nih.gov/), and the Human Protein Atlas (https://www.proteinatlas.org/).
