# Supplementary material for: High expression of cuproptosis-related SLC31A1 gene in relation to unfavorable outcome and deregulated immune cell infiltration in breast cancer: an analysis based on public databases
Source: BMC Bioinformatics. 2022 Aug 22;23:350. doi: 10.1186/s12859-022-04894-6 (PMC9394027; doi:10.1186/s12859-022-04894-6)
Supplement: Supplementary file 1 — Additional file 1: Figure S1. Differential expression analysis of 13 cuproptosis-related genes between normal and breast cancer samples. Figure S2. Kaplan-Meier plots of 4 prognosis- and cuproptosis- related genes. Figure S3. Scatter plots of correlation analysis between SLC31A1 and the other 12 cuproptosis-related genes. [file 12859_2022_4894_MOESM1_ESM.docx]

Additional file 1


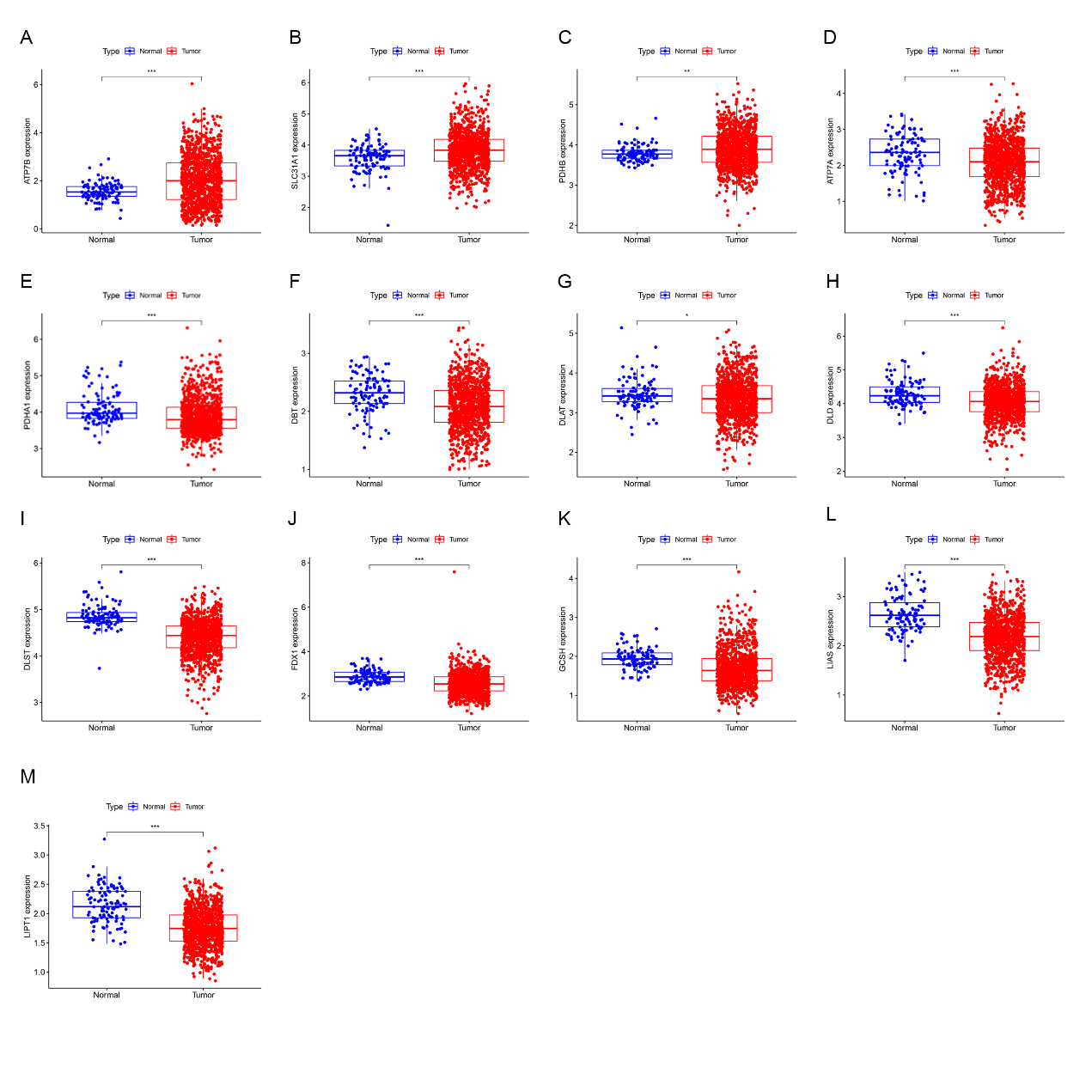


Figure S1 Differential expression analysis of 13 cuproptosis-related genes between normal and breast cancer samples.


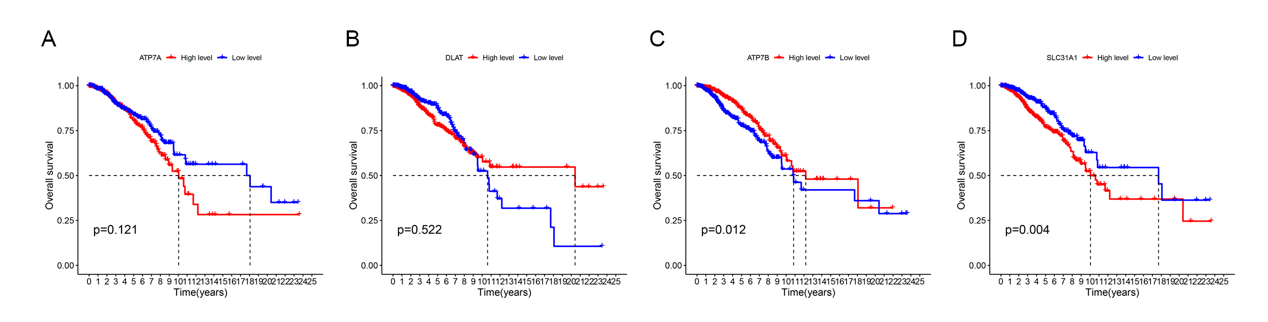


Figure S2 Kaplan-Meier plots of 4 prognosis- and cuproptosis- related genes. A-B There was no significant difference in the overall survival (OS) between the high and low gene expression groups of ATP7A or DLAT (P > 0.05), though patients with lower ATP7A expression tended to have a better OS compared with those with higher ATP7A expression. C OS of patients with higher expression of ATP7B was statistically better than those with lower gene expression (P = 0.012). D OS of patients with lower expression of SLC31A1 was statistically better than those with higher gene expression (P = 0.004).


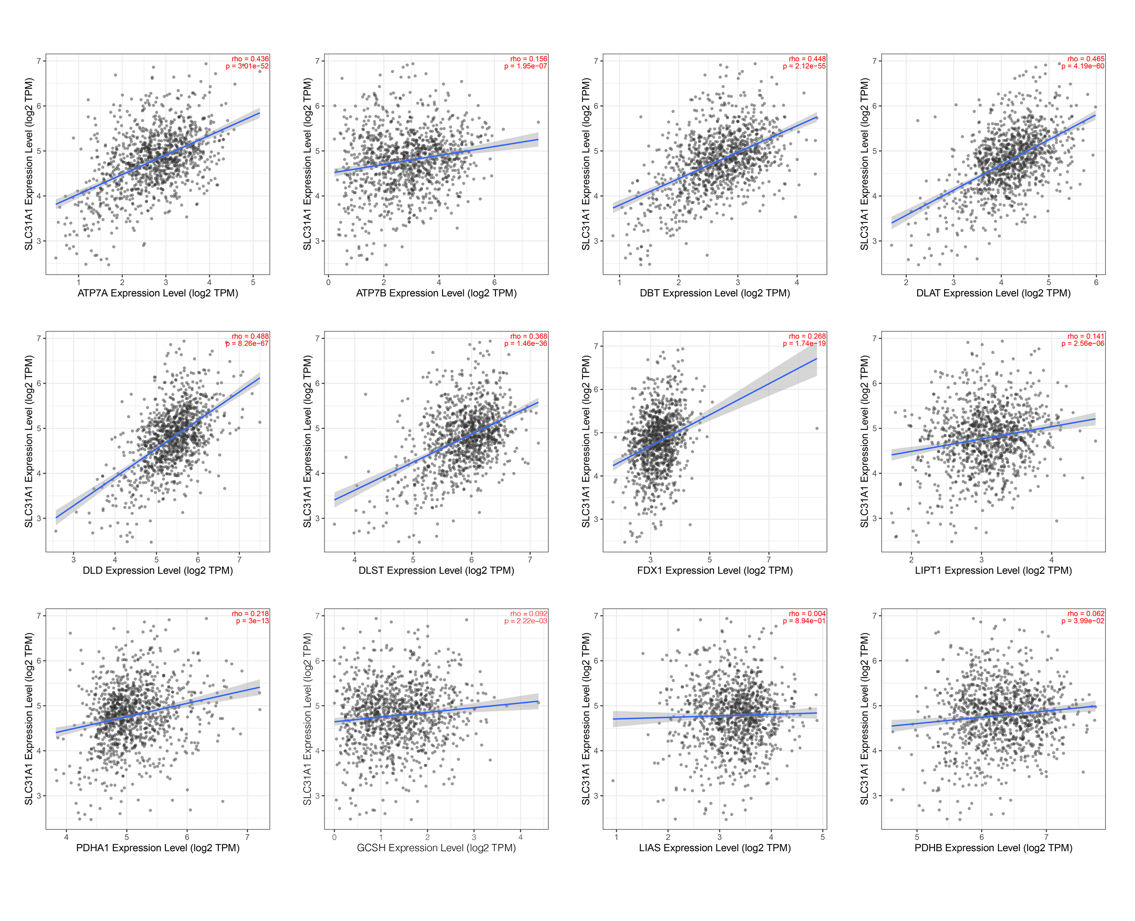


Figure S3 Scatter plots of correlation analysis between SLC31A1 and the other 12 cuproptosis-related genes. Results showed that the expression level of SLC31A1 was positively related with ATP7A, ATP7B, DBT, DLAT, DLD, DLST, FDX1, PDHB, LIPT1, GCSH and PDHA1 (P < 0.05), while there was no statistical correlation between SLC31A1 and LIAS (P > 0.05).
